# Developing a New Method of Transformation for Obtaining XYZ Color Values from RGB Images for Agricultural Applications

**DOI:** 10.3390/s24237728

**Published:** 2024-12-03

**Authors:** Vahid Mohammadi, Keivan Ansari, Pierre Gouton, Houda Attig

**Affiliations:** 1 ImViA, UFR Sciences et Techniques, Université de Bourgogne, Franche-Comté, 21078 Dijon, France; v.mohamadi1@gmail.com (V.M.); pgouton@u-bourgogne.fr (P.G.); 2 Department of Color Imaging and Color Image Processing, Color Physics Faculty, Institute for Color Science and Technology, Tehran 1668836471, Iran; 3 Research Federation of Visual and Cultural Sciences-SCV, FR CNRS 2052, 59100 Lille, France; houda.attig@univ-lille.fr

**Keywords:** color space, Munsell, Macbeth chart, transformation matrix, XYZ values

## Abstract

The extraction of device-independent color values from affordable and accessible digital images based on a standard color space system is crucially necessary for agricultural applications, where color information for plant monitoring or diagnostics is required. This study aimed to develop a transformation matrix for obtaining XYZ color coordinates from the RGB values of digital images for agricultural applications. The calibration procedure was based on Munsell and Macbeth color charts. The color coordinates of eight color charts were measured, and the transformation matrices were built. Leaf samples of six different plants were used and compared based on the proposed transformation technique. The actual XYZ values of plant leaves were measured, and the RGB values were derived from the digital images. The results indicate that the Macbeth color chart with 24 colors had the best performance, with an average ∆ELAB and CIEDE2000 of less than 1.77 and 1.97, respectively. The findings demonstrate that the proposed transformation matrix was successful in converting RGB values to XYZ values and can be employed as a quick, easy, and inexpensive technique for obtaining standard color information.

## 1. Introduction

The color information of plant leaves plays a significant role for agricultural producers [1]. This color information is used for a variety of purposes, such as monitoring plant health, disease detection, adjusting nutritional values, and estimating chlorophyll levels [2,3,4,5]. Therefore, correlating color information to physical or chemical properties is of great interest [6]. Consequently, extensive research has been conducted using the color information of plant leaves obtained from digital images, including color index estimation [7], disease detection [8,9,10,11], leaf water content estimation [12], nitrogen content estimation [13], phenotyping and identification [14,15,16], chlorophyll content estimation [17,18,19], and nutrient deficiency detection [20,21,22].

Unfortunately, in agricultural applications, precise spectrophotometers and spectrophotoradiometers are often unavailable due to the nature of farms and greenhouses, their high cost, and the lack of a stable light source. Conversely, RGB values are easily accessible, but they are device-dependent, meaning that the observed color can change when transferred between different devices (i.e., monitors, printers, etc.). To address this issue, it is necessary to find a model that expresses color independently of the device while also providing sufficient accuracy to identify the color. Research into the conversion of inexpensive color data from photographic cameras into valuable device-independent data is of great importance, particularly in the agricultural industry, where the use of expensive measuring devices is almost impossible [23,24]. In this regard, converting RGB values to a more reliable and device-independent color system, such as CIE XYZ, would facilitate accurate color measurement and its use in color discrimination.

Previous studies have introduced various techniques for extracting color and spectral information from digital color images [25]. These encompass both close-range and remote-sensing imaging techniques [26,27,28,29]. Yam and Papadakis [30] proposed a digital imaging method for measuring and analyzing the color of food surfaces. Three color models, including RGB, CMYK, and CIELAB, were employed for color information extraction, and Photoshop software (Adobe Systems, 2002) was utilized for the analyses. The advantage of their technique was that the system was quite affordable. Carney and Johnston [31] used a regression model to convert RGB image data to spectroradiometric values in CIE XYZ space for tooth-colored shades. They obtained RGB data using Image J software. The regression models yielded R^2^ values over 0.99 for RGB-to-XYZ transformation. Funt et al. [32] proposed optimal linear RGB-to-XYZ mapping for color display calibration with two methods, one using weighted least squares with weights based on the rate of change of CIELAB coordinates and the second using Nedler–Mead nonlinear optimization. They evaluated the techniques for calibrating two CRT monitors, three LCD monitors, and two LCD projectors, and the results were remarkably better than the general transformation matrix. Xiong and Funt [33] presented a nonlinear method for RGB-to-XYZ color calibration based on the technique of thin-plate splines. Their proposed technique demonstrated acceptable performance based on the analysis of a SONY camera and nine display monitors. Griesbach and Austin [34] compared Munsell and Royal Horticultural Society’s color charts in describing flower colors. It was observed that the Royal Horticultural Society’s color chart did not provide sufficient discrimination between cultivars that were distinguishable by eye, while the Munsell charts allowed for a more precise description of colors that fell within the color chart chips. Leon et al. [35] used L*a*b* measurements derived from RGB values for food science. They employed several methods to map RGB values to L*a*b* units, of which the neural network model performed best, with an error rate of 0.93%. Kumah et al. [36] used L*a*b* color coordinates from RGB images to provide color information for Fabric Patterns based on a perceptually uniform color system with respect to human color vision. Color images of printed fabrics were captured using Nikon Cameras (Tokyo, Japan), and an unsupervised segmentation method based on the mean shift algorithm was employed. The results established a consistent and reliable color measurement. Bianco et al. [37] proposed a Pattern Search Optimization (PSO) method for the conversion of RGB to XYZ values. Among the methods evaluated in their study, NonMaxIg (Non-Maximum Ignorance method) had the lowest ΔELAB of 2.78. Pegalajar et al. [38] recently used neural networks and fuzzy logic to determine the closest values for soil samples to Munsell chips. In their study, RGB images were captured using two cell phones and a professional camera. Pegalajar et al. [39] used Munsell color charts for soil colors for the classification and detection of the colors of soils. They took advantage of two machine learning techniques (i.e., ANN and fuzzy logic) for the identification of colors. The images were taken using mobile phones and one Canon camera. The proposed technique was successful, and the Canon led to higher accuracy than the mobile phone devices.

To the best of our knowledge, no reports have been presented on the extraction of the XYZ coordinates of plants derived from RGB values. Therefore, this research aims to provide a simple and accurate method for the extraction of the XYZ color coordinates of plant leaves from RGB images. This study introduces a transformation matrix based on the precise measurements of the spectral information of standard color charts.

## 2. Materials and Methods

### 2.1. Measurement Set-Up

**Figure 1:**
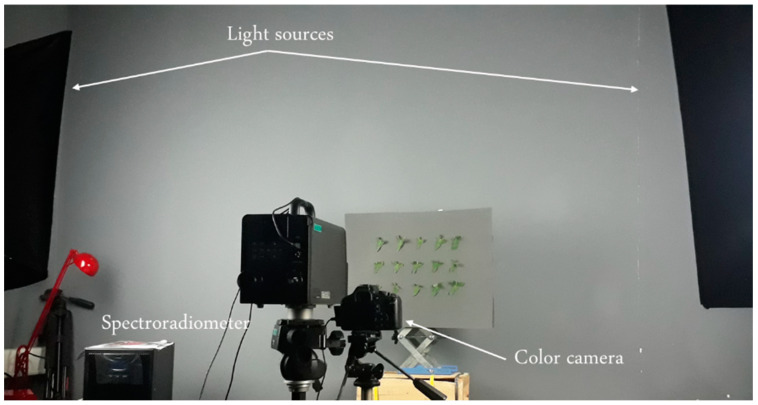
The set-up used for the measurements.

Samples of six different plants, including cucumber, eggplant, onion, pepper, sugar beet, and tomato, were collected from a farm and quickly transferred to the laboratory—including their roots and soil—in order to ensure their freshness. A total of one hundred thirty leaves from different plants were collected intact. The leaves were then placed on a grey background to capture color images and spectral data. Two D65 lights, equipped with light diffusers, were positioned at a 45° angle (Figure 1). An 18 MP Canon camera (EOS Kiss X5, Taichung, Taiwan) was utilized to take color images. A spectrophotoradiometer (Minolta, CS-2000, Tokyo, Japan) with a working range of 400 to 720 was employed to measure the spectral responses. The standard observer was set to 2°, and the spectroradiometer was positioned at 90° to the sample’s surface. Additionally, the spectral power distribution of the lights was measured using the spectrophotoradiometer, which was then used for the actual color determination.

### 2.2. RGB Values of Color Charts

**Figure 2:**
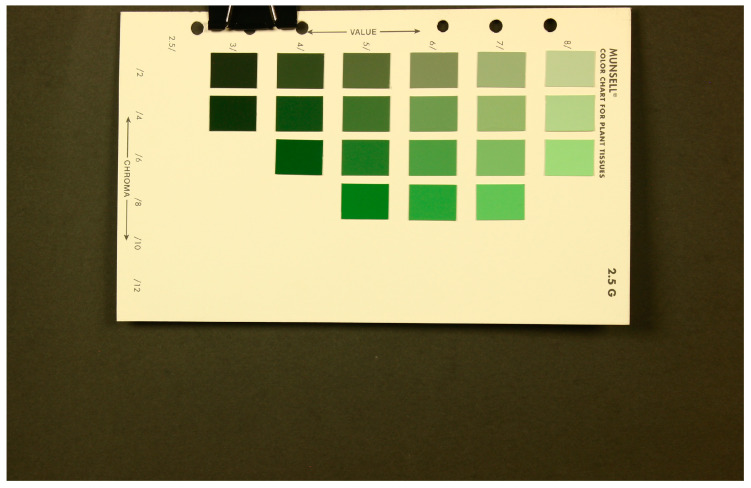
Installed color chart (e.g., Munsell 2.5G) used for capturing RGB images and measuring spectral reflectance.

**Figure 3:**
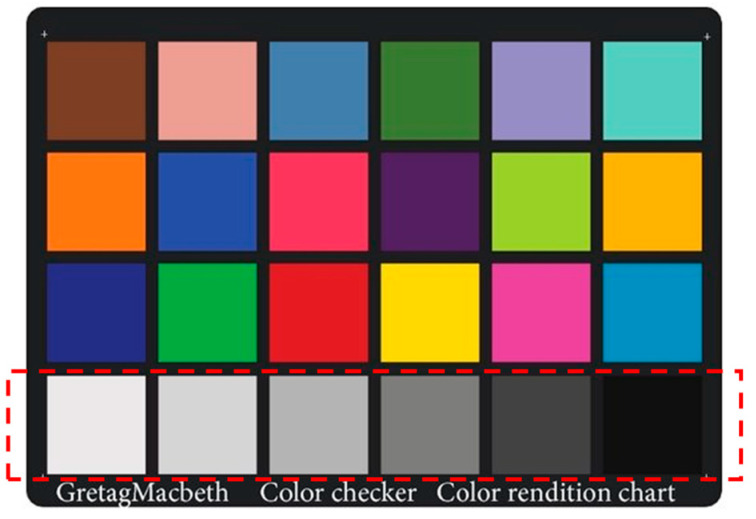
Macbeth color chart. The neutral colors are highlighted by dotted red line.

This study employed seven different color charts, namely, the Munsell color charts for agriculture in the range of 2.5G to 7.5GY and the Macbeth color chart (Table 1). The color images of all color checkers were captured (Figure 2). A code was written in MATLAB version 2019b to read the images and extract the RGB values by taking the average of one hundred pixels in the middle of each color patch. It is necessary to ensure that the response of the image sensor is linear with the XYZ tristimulus values. Hence, the RGB values were linearized by measuring the RGB and spectral reflectance responses of six neutral samples. These samples were the six samples on the last row of the Macbeth color checker, starting from white to black (Figure 3). The spectral reflectance of these neutral samples was measured. The average value was obtained for each of the patches. Then, the polynomial fits were obtained for the average values and the red, green, and blue channels separately (Equation (1)).

*p*(*x*) = *p*_1_*x^n^* + *p*_2_*x*^n−1^ + … + *p_n_x* + *p_n_*_+1_ (1)

where a third-degree polynomial was employed in this study (*n* = 3). Using the obtained polynomial transforms, the RGB values were then linearized, which ensured that the RGB values were linearly related to the XYZ tristimulus values.

### 2.3. Extraction of XYZ Coordinates of the Charts

The spectral reflectance of each color on the color charts was measured, and the XYZ and L*a*b* values were obtained for each color. The actual XYZ values were determined using the CIE formula (Equation (2)), where E(λ) represents the relative spectral power distribution of the illuminant; $\bar{x}$(λ), $\bar{y}$(λ), and $\bar{z}$(λ) are the color-matching functions for the CIE 1931 or 1964 standard observers; *P*(λ) stands for the spectral reflectance of the leaf surface; and *k* is a normalizing factor defined as $k=100/\int E(\lambda )\bar{y}\left(\lambda \right)d\lambda$ [40].

(2) $$\begin{array}{c} X=k\sum_{\lambda =360}^{\lambda =830}E\left(\lambda \right)\bar{x}\left(\lambda \right)P(\lambda )\\ Y=k\sum_{\lambda =360}^{\lambda =830}E\left(\lambda \right)\bar{y}\left(\lambda \right)P(\lambda )\\ Z=k\sum_{\lambda =360}^{\lambda =830}E\left(\lambda \right)\bar{z}\left(\lambda \right)P(\lambda ) \end{array}$$

### 2.4. Extraction of Transformation Matrices

With the XYZ and RGB values of each color on the standard charts, the transformation matrix for mapping the RGB values to XYZ values was derived for each chart. This was accomplished by calculating matrix A, which is a 3 by 7 matrix, using the equation A = T/C, where T represents the matrix of XYZ values, and C denotes the extension matrix of RGB values, which is provided as:

(3) $$C=\left[\begin{array}{c} R\\ G\\ B\\ R^{2}\\ G^{2}\\ B^{2}\\ 1 \end{array}\right]$$

The second-degree polynomial relation between XYZ and RGB values is then given by Equation (3).

(4) $$\begin{array}{c} X=a_{11}R+a_{12}G+a_{13}B+a_{14}R^{2}+a_{15}G^{2}+a_{16}B^{2}+a_{17}\\ Y=a_{21}R+a_{22}G+a_{23}B+a_{24}R^{2}+a_{25}G^{2}+a_{26}B^{2}+a_{27}\\ Z=a_{31}R+a_{32}G+a_{33}B+a_{34}R^{2}+a_{35}G^{2}+a_{36}B^{2}+a_{37} \end{array}$$

Finally, using T = AC, the XYZ values could be obtained for leaf samples from the linearized RGB values and the transformation matrices extracted for each color chart in this section. These XYZ values are the estimated values, which are based on the RGB images. The procedure for the measurement of the actual XYZ values of leaf samples is explained in the next section. The actual XYZ values were obtained for the calculation of the precision of the RGB-to-XYZ transformation matrices.

### 2.5. Color Coordinates of Plant Leaves

**Figure 4:**
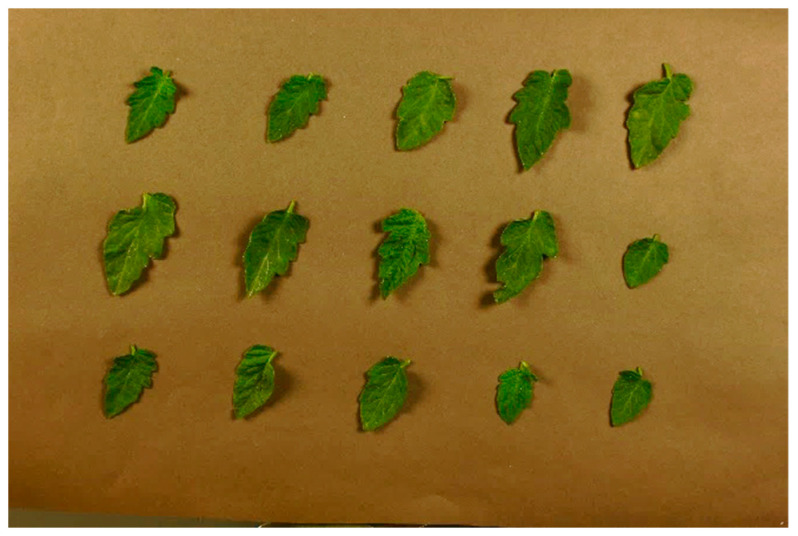
Installed leaves for taking the RGB image and measurement of spectral reflectance.

For the plant leaves, the actual XYZ values were determined by spectral reflectance using the same method as the color charts. For the extraction of RGB values, an area in the middle of the leaves containing over 100 pixels was chosen, and the average values of the pixels were obtained for each layer of the image to be selected as RGB values. Subsequently, linearization was performed on the RGB values, and the XYZ values were calculated from the linearized RGB values using the CIE XYZ color-matching functions. Figure 4 illustrates the installation of leaves for the acquisition of an RGB image and the measurement of spectral reflectance.

For comparison of the extracted XYZ values with the classical method, the XYZ coordinates of the leaves were extracted using a general conversion matrix as follows [41]:

$$\left[\begin{array}{c} X\\ Y\\ Z \end{array}\right]=\left[\begin{array}{ccc} 0.4124 & 0.3576 & 0.1805\\ 0.2126 & 0.7152 & 0.0722\\ 0.0193 & 0.1192 & 0.9505 \end{array}\right]\left[\begin{array}{c} R\\ G\\ B \end{array}\right]$$

### 2.6. Color Difference Calculation

The XYZ values were then converted into CIELCH values, a color space based on CIE L*a*b*. CIELCH uses the polar coordinates C* (i.e., chroma and relative saturation) and h° (i.e., the angle of the hue in the CIELAB color space) instead of the Cartesian coordinates of a* (red-green) and b* (yellow-blue). The conversion is performed in two steps: first, XYZ to L*a*b* and then L*a*b* to L*c*h° values.

(5) $$\begin{array}{c} C^{*}=\sqrt{{\left(a^{*}\right)}^{2}+{\left(b^{*}\right)}^{2}}\\ h=atan2(a^{*},b^{*})\\ h^{{}^{\circ}}=\left(\frac{h\times 180}{\pi }\right)mod\;360 \end{array}$$

The color difference, ∆E, between the actual and calculated L*c*h° color values was then obtained using the CIEDE2000 (∆E_00_) formula. This formula provides more perceptual uniformity than ∆ELAB1976 and is expressed as follows:

(6) $${∆E}_{00}=\sqrt{{\left(\frac{{∆L}^{\prime }}{k_{L}S_{L}}\right)}^{2}+{\left(\frac{{∆C}^{\prime }}{k_{C}S_{C}}\right)}^{2}+{\left(\frac{{∆H}^{\prime }}{k_{H}S_{H}}\right)}^{2}+R_{T}\frac{{∆C}^{\prime }}{k_{C}S_{C}}\frac{{∆H}^{\prime }}{k_{H}S_{H}}}$$

where the differences in lightness, chroma, and hue, are denoted as ${∆L}^{\prime }$, ${∆C}^{\prime }$, and ${∆H}^{\prime }$. To compensate for the lightness, chroma, and hue components of CIELCH, $S_{L}$, $S_{C}$, and $S_{H}$ are employed as compensation functions, respectively. Parametric weighting factors, $k_{L}$, $k_{C}$, and $k_{H}$, are also used to adjust the importance of each component. Additionally, a hue rotation term, ($R_{T}$), is included to address the problematic blue region.

## 3. Results and Discussion

In this study, Munsell color charts for agricultural applications, the Macbeth color chart, and a traditional conversion method were compared to identify the most suitable chart for determining the color of plants for agricultural applications. This provides the utility of a color chart for the online extraction of colorimetric properties of leaves using common RGB cameras, as well as the application of this information for the estimation of other biological and chemical properties of plants.

**Figure 5:**
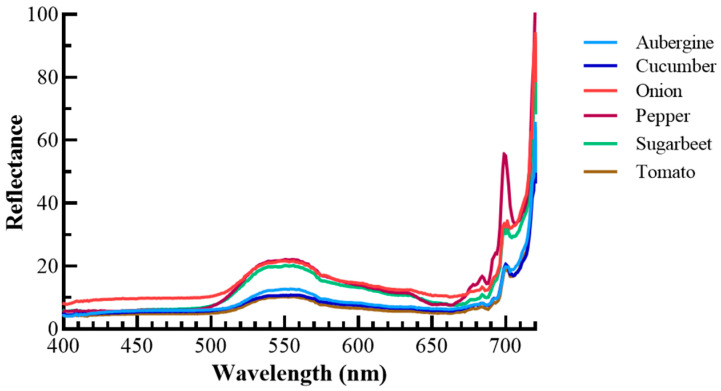
Average spectral reflectance of different plants in the visible range.

Spectral reflectance was measured in steps of one nanometer to obtain all the reflected energy, providing the precise color information of the leaves. Figure 5 represents the spectral reflectance of different plant leaves. As expected, most of the reflection occurs in the green area; however, in the red area of the spectra, there exists much information that is crucial for the extraction of precise color values.

### 3.1. Estimation of XYZ Coordinates

Table 2 presents the CIELab and CIEDE2000 color differences of the actual and estimated XYZ values based on the transformation matrices obtained from the color charts. The results indicate that for almost all plants, the Macbeth color chart yielded the least amount of ∆E. Only for eggplant, Munsell 7.5GY has the smallest ∆E. The best estimation was achieved for the pepper plant, having a CIELab of 1.07 and CIEDE2000 of 0.86. In all cases, the Macbeth color chart led to an acceptable ∆E, especially using the CIEDE2000 technique, which presented smaller values than CIELab. Munsell 5GY and Munsell 7.5GY led to color differences between 1 and 3, which represent small color differences, noticeable by eye but still in the range of acceptable color differences. On the other side, the general conversion matrix led to color differences bigger than 3, representing large color differences.

As mentioned, among Munsell color charts, 5GY and 7.5GY present small errors for estimation purposes. On the other side, Munsell 2.5 shows the worst estimations with very large ∆Es. Also, it is observed that the obtained specific transformation matrices based on Munsell charts (such as 5GY and 7.5GY) have errors far smaller than the general conversion matrix that traditionally is used for RGB-to-XYZ transformation. Fan et al. [42] evaluated smartphone cameras to compare them with Munsell color charts. They transformed the RGB values to CIELAB coordinates for comparison. The ∆E color differences for both the camera and Munsell charts were in a range of 4 to 15. In a recent work, Berndt and Gaussoin [43] linked the color of turfgrass leaves to colorimetric coordinates (i.e., hue, value, and chroma) of the Munsell Plant Tissue Color Book. They used different equations for each coordinate and obtained acceptable error values (less than 0.1%). Their study also showed that the colorimetric coordinates of plants can be specified and communicated.

Table 3 provides the average values of XYZ coordinates for the actual values and the estimated values based on the Macbeth chart. The standard deviation values of the repetitions and the color differences have also been provided. It is observed that the proposed specific transformation matrix leads to quite precise estimations. As the table shows, the obtained XYZ values are remarkably near the actual values. It is noted that the standard deviation of the estimated XYZ values is quite similar or even less than the ones for the actual XYZ values. The best estimation is observed for the pepper plant, amounting to ∆E of 0.86, which is considered not noticeable by eye. It is observed that the average ∆E for all plants is 1.44, which indicates a relatively good color transformation.

### 3.2. XYZ Visualization on CIE Chromaticity Diagram

**Figure 6:**
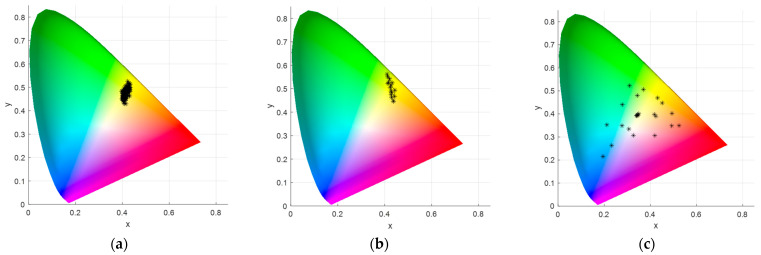
The distribution of the obtained XYZ values on the CIE chromaticity diagram; (**a**) for plant leaves, (**b**) for Munsell 5GY, and (**c**) for MacBeth color chart. The figure represents the area of the chromaticity diagram covered by XYZ values.

To visually compare the XYZ values of the leaves and charts, the distribution of the XYZ values of plant leaves, the 5GY Munsell chart, and the McBeth color chart were developed on a chromaticity diagram of CIE (Figure 6). As the figure represents, the XYZ values of the Munsell chart are quite close to the ones of plants; however, it does not lead to the best estimation. This normally happens as the covered zone of the chart does not cover all of the zones enclosing the colors of leaves. On the other side, the Macbeth chart, which covers a remarkable zone of the CIE diagram, leads to the best RGB-to-XYZ transformation. In other words, it covers a larger zone of the chromaticity diagram, and this might have led to a better model of the RGB-to-XYZ transformation.

### 3.3. Color Values of the Macbeth Chart

Table 4 displays the measured RGB and XYZ values for the Macbeth color chart. This result shows that the specific transformation obtained in this work can provide a proper estimation of the XYZ values. Table 5 illustrates the transformation matrix obtained based on the Macbeth color chart.

The results of this study provide a good estimation of the XYZ values as the main colorimetric information based on RGB images is accessible and available to everybody. This will make it possible to easily identify colorimetric information. The extraction of XYZ values directly, which includes the use of colorimetric systems or spectrophotometers, has been reported [44,45,46,47,48]. Moreover, this study shows that this technique is quick, inexpensive, accessible, and adaptable to greenhouses and farms. The adaptation of colorimeters or spectrophotometers for the measurement of colorimetric coordinates can be achieved as well. Sanmartín et al. [49] studied the measurement of the colorimetric coordinates of small plant samples by proposing a method based on cardboard adaptors on two aperture masks of a portable spectrophotometer.

## 4. Conclusions

This work aimed at the estimation of the XYZ coordinates of leaf color for six different plants based on inexpensive and accessible digital images. Several transformation matrices based on eight different color charts were obtained and compared. This study demonstrates that the transformation matrix obtained from the Macbeth color chart provided the best transformation of RGB to XYZ values for plant samples. The results obtained from the Macbeth color chart show a very precise transformation, with standard color differences of less than 1.97 based on ∆E2000, while the conventional general matrix resulted in color differences over 4.5 for different plants. Also, Munsell color charts 5GY and 7.5GY led to acceptable color differences. Among the Munsell color charts, 5GY provided the best transformation of RGB to XYZ values. It was observed that the proposed transformation matrices based on Macbeth and Munsell charts lead to more accurate estimations compared to the general transformation matrix. Therefore, the proposed transformation matrix is a proper solution for the extraction of color information in agricultural applications to provide an inexpensive, quick, and reliable technique for the estimation of XYZ coordinates of plant leaf color.

## Figures and Tables

**Table 1:** List of color charts used for extraction of transformation matrix.

	Name/Number	Number of Colors
Munsell color chart for plant tissues	2.5G	20
	2.5GY	20
	5BG	14
	5G	17
	5GY	16
	7.5G	16
	7.5GY	23
Macbeth color chart	Color rendition chart	24

**Table 2:** Average values of ∆E CIELAB and CIEDE2000 for the original and predicted XYZ values based on different color charts.

	Cucumber		Eggplant		Onion		Pepper		Sugar beet		Tomato	
Color Chart	∆ELab	CIEDE2000	∆ELab	CIEDE2000	∆ELab	CIEDE2000	∆ELab	CIEDE2000	∆ELab	CIEDE2000	∆ELab	CIEDE2000
Munsell 5BG	3.03	3.06	3.00	3.22	2.86	2.63	3.73	3.46	3.73	3.48	2.98	3.15
Munsell 2.5G	5.19	6.32	4.73	5.85	3.87	4.19	5.47	5.95	4.65	4.78	5.69	7.17
Munsell 5G	5.13	5.50	4.79	5.15	4.24	4.09	5.43	5.61	4.54	4.68	5.56	6.13
Munsell 7.5G	4.68	4.39	4.54	4.52	3.16	2.99	3.63	3.61	3.89	3.80	5.05	4.79
Munsell 2.5GY	73.48	65.00	56.70	44.26	40.63	23.53	33.66	19.94	37.85	23.02	73.67	64.18
Munsell 5GY	2.91	2.57	2.21	1.74	2.40	1.69	2.00	1.45	3.01	2.26	2.62	2.17
Munsell 7.5GY	3.57	2.97	1.73	1.43	2.28	1.69	2.45	1.90	3.89	2.79	2.55	2.20
Macbeth chart	1.86	1.77	1.74	1.64	1.43	1.10	1.07	0.86	1.97	1.58	1.86	1.69
General matrix	5.48	4.56	5.59	4.70	5.65	4.72	6.13	5.16	5.53	4.57	6.32	6.24

**Table 3:** Average values of color coordinates obtained using the general and proposed conversion matrices.

Plant	Actual Values			Estimated Values			
	X	Y	Z	X	Y	Z	∆E2000
Cucumber	8.17 ± 2.14	9.01 ± 2.40	2.67 ± 0.58	8.29 ± 2.15	9.64 ± 2.47	2.61 ± 0.32	1.77
Eggplant	8.99 ± 1.83	10.21 ± 2.02	2.85 ± 0.50	9.41 ± 1.18	11.16 ± 1.17	2.76 ± 0.43	1.64
Onion	15.74 ± 2.85	17.68 ± 3.11	4.81 ± 1.10	11.77 ± 3.23	14.24 ± 3.55	3.13 ± 0.84	1.10
Pepper	15.04 ± 4.19	17.51 ± 4.47	3.00 ± 1.04	14.70 ± 3.54	17.40 ± 3.70	3.09 ± 0.84	0.86
Sugar beet	14.03 ± 3.20	16.19 ± 3.26	3.03 ± 0.94	16.47 ± 5.23	19.23 ± 5.37	3.88 ± 1.60	1.58
Tomato	7.54 ± 1.57	8.55 ± 1.46	2.46 ± 0.57	8.12 ± 0.85	9.56 ± 0.85	2.48 ± 0.26	1.69

**Table 4:** Measured RGB and XYZ values of the Macbeth chart patches.

No.	Color Name	R	G	B	X	Y	Z
1	Dark skin	40.715	19.932	7.843	14.015	11.452	3.304
2	Light skin	142.105	80.072	72.139	47.207	37.287	11.082
3	Blue sky	44.456	56.353	85.221	17.697	17.789	15.487
4	Foliage	30.563	43.947	6.83	12.703	14.737	3.267
5	Blue flower	68.302	64.338	108.169	25.675	22.793	19.663
6	Bluish green	71.314	138.944	112.542	31.415	39.694	19.058
7	Orange	168.558	60.976	11.362	50.874	36.332	3.088
8	Purplish blue	28.79	35.618	96.506	12.318	10.721	17.728
9	Moderate red	127.06	29.738	40.296	38.35	23.759	6.0848
10	Purple	24.963	10.327	35.465	8.915	6.902	6.67
11	Yellow green	106.278	132.158	29.337	39.913	45.917	4.892
12	Orange green	172.172	102.714	14.722	57.653	49.495	3.46
13	Blue	9.58	15.383	70.005	6.309	5.133	12.388
14	Green	40.627	81.672	23.241	16.956	23.333	4.363
15	Red	83.234	7.314	7.453	26.225	15.421	2.473
16	Yellow	193.399	149.229	30.532	72.057	67.502	4.203
17	Magenta	110.476	30.323	84.634	34.178	21.391	14.285
18	Cyan	22.154	69.766	106.333	13.169	16.50	17.114
19	White	206.599	204.274	201.111	93.589	89.349	40.112
20	Neutral 8	152.000	148.728	149.521	62.115	59.369	28.256
21	Neutral 6.5	99.383	96.282	96.719	37.446	35.63	17.299
22	Neutral 5	53.804	52.27	49.446	20.095	19.184	9.107
23	Neutral 3.5	22.153	20.886	18.706	9.20	8.821	4.416
24	Black	6.421	6.295	7.497	3.598	3.428	1.719

**Table 5:** Transformation matrix obtained for Macbeth color chart.

0.2446	0.0060	−0.0100	0.0002	0.0005	0.0003	3.0619
0.1175	0.1465	−0.0313	0.0002	0.0006	0.0002	2.9859
0.0045	0.0101	0.1058	−3.9546	−0.0001	0.0005	1.7726

## Data Availability

Data is contained within the article and Supplementary Materials.

## Supplementary Materials

The following supporting information can be downloaded at: https://www.mdpi.com/article/10.3390/s24237728/s1.
